# An Important Announcement

**Published:** 1916-03

**Authors:** 


### An Important Announcement.

It is a pleasure to be able to announce to the readers of the TEXAS MEDICAL JOURNAL a series of articles by Dr. Henry R. Harrower, now of Los Angeles, Cal., on "The Diagnosis of the Internal Secretory Disorders." This will commence in the issue for May, and will take up this increasingly important subject from the standpoint of the general practitioner.

The part that the ductless glands play in health and disease is none too well appreciated as yet, and many a puzzling condition is made clear when the influence of the hormones is understood. There is practically no convenient literature upon the all-important diagnostic phase of this subject. True, there are hundreds of papers in a dozen languages dealing with the pathology and physiology, prognosis and treatment of the better known endocrinous diseases; but it is no easy task to collate from these the essential, practical points which make for success in our work. This has been done in this series, and done extremely well; and we are confident that our readers will keenly appreciate this innovation.

There will be ten or more concise and practical articles. A "Question-Clinic" will be arranged for that will be open to all paid-up subscribers, and this will make the subject of the most possible practical value to all.

How about your subscription?

